# “But I did not touch nobody!”—Patients' and nurses' perspectives and recommendations after aggression on psychiatric wards—A qualitative study

**DOI:** 10.1111/jan.14107

**Published:** 2019-07-10

**Authors:** Jentien M. Vermeulen, Paul Doedens, Lindy‐Lou N. J. Boyette, Bea Spek, Corine H. M. Latour, Lieuwe de Haan

**Affiliations:** ^1^ Psychiatry, Amsterdam UMC (location Academic Medical Center) University of Amsterdam Amsterdam The Netherlands; ^2^ ACHIEVE Centre for Applied Research, Faculty of Health Amsterdam University of Applied Sciences Amsterdam The Netherlands; ^3^ Department of Clinical Psychology University of Amsterdam Amsterdam The Netherlands; ^4^ Clinical Epidemiology, Biostatistics and Bioinformatics, Amsterdam UMC (location Academic Medical Center) University of Amsterdam Amsterdam The Netherlands; ^5^ Arkin Amsterdam The Netherlands

**Keywords:** aggression, mental health, nursing, patient safety, perspective, psychiatry, violence

## Abstract

**Aims:**

To gain a deeper understanding of the differences in patients and staff perspectives in response to aggression and to explore recommendations on prevention.

**Design:**

Qualitative, grounded theory study.

**Methods:**

We conducted semi‐structured interviews with patients and nurses involved in an aggressive incident. Data collection was performed from May 2016 ‐ March 2017.

**Results:**

Thirty‐one interviews were conducted concerning 15 aggressive incidents. Patients and nurses generally showed agreement on the factual course of events, there was variation in agreement on the perceived severity (PS). Patients' recommendations on prevention were mostly personally focussed, while nurses suggested general improvements.

**Conclusion:**

Patients are often capable to evaluate aggression and give recommendations on prevention shortly after the incident. Patients and nurses differ in the PS of aggression. Recommendations on prevention of patients and nurses are complementary.

**Impact:**

What problem did the study address? Perspectives of patients and nurses differ with respect to aggression, but how is unclear. What were the main findings? Patients and nurses generally described a similar factual course of events concerning the incident, patients often perceive the severity less than nurses. Patients are capable to give recommendations on prevention of aggressive incidents, shortly after the incident. Where and on whom will the research have impact? Factual course of events can be a common ground to start evaluating aggressive incidents and post‐incident review should address the severity of incidents. Asking recommendations from patients on how to improve safety and de‐escalation can lead to innovative and personal de‐escalation strategies and supports patients autonomy.

## Abstract (zh)

**目的:**

深入理解病人与护士对待侵略性事件的观点的不同,探索防止侵略性事件的建议。

**设计:**

定性的、基于理论的研究。

**方法:**

我们对涉及侵略性事件的病人和护士进行半结构式采访,并在2016年5月‐2017年3月期间进行数据收集。

**结果:**

对涉及15起侵略性事件的31名病人和护士进行访谈。病人与护士对事件经过的事实上观点一致,对事件严重程度的感知不一致。病人关于预防侵略性事件的建议比较偏向个人方面的预防,护士关于预防侵略性事件的建议主要是综合性预防。

**结论:**

病人能够评价侵略性事件并能在事件发生后立即给出预防建议,病人与护士对事件的严重成都的感知不一致,但两者提出的意见互补。

**影响:**

此研究是为了解决什么问题?病人与护士对侵略性事件的观点不同,不同在什么地方?此研究的主要发现是什么?与护士对事件经过的事实上观点一致,但病人感知的事件严重程度比护士感知到的严重程度低。病人能在事件发生后立即给出预防建议。此研究将在何处对何人造成影响?事件经过可以作为评价事件的共同基础。事后回顾主要研究事件严重程度。向病人询问关于提升环境安全和防止事态扩大的建议,会探索出创新的个性的建议同时也维护了病人的自主权。

## 1 INTRODUCTION

Aggressive behaviour is a worldwide problem in healthcare (Gaynes et al., 2017; Rubio‐Valera et al., 2015). Nurses working in closed psychiatric units are at high risk for experiencing physical violence at work (Campbell et al., 2011). The danger of aggression is the main reason for professionals on psychiatric wards to apply coercive measures, such as seclusion and restraint (Cullen et al., 2016; Kallert et al., 2005). Coercive measures may threaten the therapeutic alliance between patients and professionals (Höfer, Habermeyer, Mokros, Lau, & Gairing, 2015). The international mental health community aims to ban coercive measures from practice (Vruwink, Mulder, Noorthoorn, Uitenbroek, & Nijman, 2012). To prevent the use of coercive measures, it is important to gain insight in perspectives of patients and staff on aggressive incidents and tailored recommendations concerning prevention are needed.

### 1.1 Background

Several studies evaluated the perspective of patients after aggressive incidents (Gudde, Olso, Whittington, & Vatne, 2015; Kontio et al., 2014, 2012). Other studies reported on perspectives and attitudes of staff members towards aggression (Jansen, Dassen, & Groot Jebbink, 2005). Several studies investigated experiences and perspectives of patients and staff members on aggressive incidents in general (Dickens, Piccirillo, & Alderman, 2013; Duxbury, 2002; Duxbury & Whittington, 2005; Gillig, Markert, Barron, & Coleman, 1998; Hallett, Huber, & Dickens, 2014; Pulsford et al., 2013). Some found considerable consensus between patients and nurses in their perspective (Dickens et al., 2013; Duxbury, 2002; Pulsford et al., 2013). Others found major differences, especially concerning perspectives on the nature and cause of aggressive incidents (Duxbury & Whittington, 2005; Gillig et al., 1998; Lamanna et al., 2016). These studies lacked triangulation of different perspectives on the same incident. To our knowledge, two studies did triangulate the perspective of patients and staff members. Omerov, Edman, and Wistedt (2004) used a questionnaire for staff members and Ilkiw‐Lavalle and Grenyer (2003) tested differences in categories from questionnaires, to compare the experiences of patients and staff of a specific aggressive incident. Both found several differences in perspectives concerning causes (i.e. patients perceived environmental factors important as cause for aggression, while staff focussed on the patient's illness) and potential prevention of the incident (i.e. inability of staff to identify patients' provocations for aggression). These differences are believed to influence patient‐staff interaction (Dickens et al., 2013; Duxbury, 2002; Duxbury & Whittington, 2005) and may affect recommendations to prevent aggression (Hallett et al., 2014). The existing literature described overall differences in perspectives and recommendations, but the nature of these differences in perspectives of the persons involved remains unclear. To use the knowledge on these differences for prevention of aggression, a more thorough understanding of the differences in perspective is crucial. Additionally, further insight into similarities and differences between patients and nurses concerning recommendations is essential to use these recommendations effectively in the prevention of patient violence.

## 2 THE STUDY

### 2.1 Aims

The aim of this study is to gain deeper understanding in the differences in perspective between patients and nurses concerning a specific aggressive incident. We addressed the following research questions: (a) What is the underlying theory on the differences and similarities of the view on aggressive incidents? (b) Which recommendations are provided to prevent aggressive incidents in the future?

### 2.2 Design

We used a grounded theory design to identify the underlying concepts to provide a theoretical explanation based on narrative data (Corbin & Strauss, 1990). This study is reported according to the Consolidated Criteria for Reporting Qualitative Studies (COREQ; Tong, Sainsbury, & Craig, 2007; Supplement 1).

### 2.3 Sample/participants

A convenience sample of nurses and patients who were involved in an aggressive incident was recruited. An aggressive incident was defined as: “any verbal, nonverbal or physical behaviour that was threatening (to self, others, or property), or physical behaviour that actually did harm (to self, others, or property)” (Morrison, 1990).

Recruitment started with a presentation at the ward about the aims and procedures of our study. We aimed at including both more and less severe incidents, to collect data that is as rich as possible. Nurses were asked to report aggressive incidents to the researchers through email. Immediately after receiving a report, one of the authors (JV) came to the ward to approach the nurse and patient for study participation. Inclusion criteria for patients and nurses were being a participant in an aggressive incident and willing to participate in an interview. Exclusion criteria for patients were a severe language barrier, current stay in seclusion or previous participation in the current study.

The setting was a 12‐bed closed psychiatric ward for adults of a university hospital in The Netherlands that admits approximately 150 patients annually. The closed admission ward provides acute psychiatric care for patients with various diagnoses, mostly psychotic disorders and mood disorders. Reasons for admission always include (potential) danger due to the psychiatric disorder. Most of the patients (>80%) are admitted involuntary in the context of the Dutch civil Mental Health Act (BOPZ, 1992).

The wards' team consists of 25 registered nurses, educated on European Qualifications Framework level four (secondary vocational education) or six (bachelors' degree). Additional training in verbal de‐escalation and physical restraint is part of the ward's routine training program. Verbal de‐escalation is an intervention that consists of calmly managing an agitated client to prevent (further) violence (Mavandadi, Bieling, & Madsen, 2016).

During the study period, 22 aggressive incidents were reported. This is an underestimation of the number of aggressive incidents on the ward, probably due to under‐reporting. Under‐reporting of aggressive incidents is a well‐known problem in healthcare (Taylor & Rew, 2011). The authors assume that nurses only reported more severe aggressive incidents, because a relatively high number of patients in our study were secluded after the incident.

Seven eligible patients declined to participate, mostly because of lack of interest or distrust regarding audiotaping their comments. None of the patients were excluded because of their psychiatric condition or language barrier. None of the nurses declined to participate. This resulted in a sample of 15 unique patients and 13 nurses representing 15 unique aggressive incidents, with a total of 31 interviews. One incident had two nurses involved who were both interviewed and three nurses were involved in more than one aggressive incident.

### 2.4 Data collection

Data collection was performed from May 2016 ‐ March 2017. Semi‐structured interviews were conducted in a private room at the ward to enhance confidentiality. One of the first authors (JV) performed the interviews because she was not part of the ward's treatment team. The interviews were planned short after the incident aiming to capture vivid memories from the patient while being in comparable psychiatric state as during the incident. In case of seclusion, patients were approached shortly after termination of the seclusion episode. Planned duration of the interviews was approximately 15 min, to diminish potential burden for patients. Patients who were not included or declined participation, were approached by nursing staff for post‐incident review, in line with regular practice.

During the interviews, a topic list was used with questions that had been developed with an experienced qualitative researcher, tested for face validity with an expert by experience and pilot tested in three interviews. The following questions were asked: (a)* Can you describe the aggressive incident that you have recently been through?* (b)* Can you describe the response of the staff and your opinion about this response? *(c)* Can you give any suggestion that could have prevented the aggressive incident and/or improved the care at that time?*

The interviewer stimulated participants to give in‐depth information about the factual course of events during the incident, the acts of nurses during the incident and their recommendations on prevention. Interviews were digitally audiotaped. We decided not to make field notes, because the participating patients often suffered from paranoia and making notes could induce suspicion and agitation.

### 2.5 Ethical considerations

This study was reviewed by the Medical Ethics Review Board of our institution which decided that formal approval was not necessary (Supplement 2). The Dutch Medical Research (Human Subjects) Act (WMO) states that formal ethical approval is necessary when the study meets two criteria: (a) It concerns medical/scientific research; and (b) participants are subject to procedures or are required to follow rules of behaviour (WMO, 1992). The primary reason that the Medical Ethics Review Board decided that our study was not considered as a study within the influence of the WMO is that we only investigated usual patient care. Debriefing aggressive incidents is considered regular clinical practice. Therefore, in our study subjects were not required to follow rules of behaviour beyond normal clinical practice. The WMO is based on international quality standards for medical research, such as the declaration of Helsinki and Good Clinical Practice.

The researchers approached eligible patients and explained the objective of the study, the goal and duration of the interview and the right to refuse and to withdraw consent at any time. Even severely unwell inpatients in mental health care can be capable of decision‐making for research (Spencer, Gergel, Hotopf, & Owen, 2018). We gave considerable attention to informing patients on the nature of the study and their rights to refuse or withdraw consent, as recommended in earlier research on obtaining informed consent from inpatients in mental health care (Carpenter et al., 2000). We obtained written or audiotaped informed consent from all participating patients. Information from the interview was not discussed with the patients' treatment team. Thereby, the researchers had no influence on clinical decisions. The participation of staff members was not discussed with the departments' management team or with their co‐workers. The privacy of all participants was protected according to the Dutch privacy protection legislation.

### 2.6 Data analysis

Interviews were transcribed verbatim in MAXQDA version 12. The two first authors of this study (both PhD‐students) independently analysed all transcripts after receiving additional training in performing qualitative research. This started after the first interview and was performed alongside with the data‐collection. First, we carefully read the transcripts to become familiar with the data. Subsequently, during re‐reading, the content was coded in‐vivo and afterwards codes were clustered into concepts (coding tree available on request). Quotes for this manuscript were selected during consensus meetings.

Regarding the perspective of patient and nurse, incidents were analysed in patient‐nurse dyads. We carefully read the codes again and independently rated whether overlap of perspectives between nurse and patient was found. The first authors held several consensus meetings with the last author to discuss the concepts that were identified and the core category in the data (Heath & Cowley, 2004). During analysis, the authors went back and forth to the data to verify emerging concepts.

Because there is a substantial body of evidence regarding recommendations on prevention of aggression (Gillig et al., 1998; Gudde et al., 2015; Hallett et al., 2014; Kontio et al., 2014, 2012; Meehan, McIntosh, & Bergen, 2006), we followed a slightly different procedure analysing this research question. Recommendations were analysed independently of specific incidents. After reading, coding and clustering the codes into concepts, three researchers (JV, PD and LdH) discussed the content of the interviews after every two or three incidents and decided if new concepts of recommendations emerged.

Transcripts and results were not returned to the participants, because of the vulnerable patient population. For publication, Dutch quotes were translated to English by one of the authors (LLB) who was raised bilingually. Translation was as literal as possible to stay close to the words used by participants. This results in some grammatically incorrect sentences and in some cases in a choice of words that is somewhat erratic.

## 3 FINDINGS

Sociodemographic variables of patients are presented in Table 1. Nurses who participated in this study were all registered nurses, six were male and seven female. The interviews lasted from 8 to 25 min and were conducted a median of 3 days after the incident.

**Table 1:** Sociodemographics of the included patient sample (*N* = 15)

Variables	*N*(%)
Types of aggression	
Verbal aggression	3 (20)
Physical aggression to others	3 (20)
Physical aggression to objects	9 (60)
Gender male/female	10 (67)/5 (33)
Age, median (IQR)	28 (26–37)
Primary diagnosis	
Psychotic disorder^a^	10 (67)
Bipolar I disorder	4 (27)
Other^b^	1 (6)
Compulsory admission	15 (100)
Concluding of incident	
Seclusion	12 (80)
Time out	3 (20)
Incidents concluding with restraint	0
Length of admission, days, median (IQR)	75 (52–180)
Number of days between incident and interview, median (range)	3 (2–13)

Abbreviations: IQR, interquartile range; *SD*, standard deviation.

a Psychotic disorder: schizophrenia, schizoaffective, due to medical disorder.

b Personality disorder.

### 3.1 Concepts

Two concepts emerged from our data regarding the perspective of patients and nurses on aggressive incidents, namely *facts* (the factual course of events of the provocation, escalation and solution of the aggressive incident) and *subjective experience*. The major difference between patients and nurses is found in the latter, particularly in the perceived severity (PS) of the incident. We identified PS as our core category of the difference in perspectives.

The core category that emerged from our data regarding recommendations was that there were distinct “patients' recommendations” and “nurses' recommendations”. Patients gave recommendations on their own treatment, while nurses tended to give recommendations on the de‐escalation of aggressive incidents in general. Furthermore, there were five subthemes emerging from patients' recommendations, namely: (a) humane treatment and freedom; (b) ward routine; (c) interpersonal contact; (d) personalized de‐escalation interventions; and (e) shared decision making during a coercive measure. Subthemes that emerged from the nurses' recommendations were: (a) pharmacological interventions; (b) timing of interventions; (c) and facility related factors.

### 3.2 Perspectives

#### 3.2.1 Factual course of events

We observed high similarity in the factual course of the aggressive incident between patients and nurses. Both described similar facts (such as place of the incident and length of the intervention) of the incident. The facts prior to the incident and in the last phase of the incident (i.e. intervention) showed most resemblance. A brief description of patients and nurses for each incident can be found in Table 2.

P13: On that moment? I get angry and start screaming: I want help needed. Bring me my doctor. I want to see my doctor!

N13: Then we offered paracetamol and other things for the pain, ehm, she was actually really agitated and demanding and “A doctor must come now!”

**Table 2:** Description of involved patients and nurses

Incident	Involved patient	Sex, native language (interview language^a^)	Involved nurse(s)	Sex, native language (interview language^a^)
I1	P1	Male, Dutch	N1	Male, Dutch
I2	P2	Male, Dutch	N2	Female, Dutch
I3	P3	Male, Dutch	N3 N4	Male, Dutch Female, Dutch
I4	P4	Male, Dutch	N5	Male, Dutch
I5	P5	Male, Italian (English)	N6	Female, Dutch
I6	P6	Male, Dutch	N7	Female, Dutch
I7	P7	Male, German (English)	N8	Female, Dutch
I8	P8	Male, Dutch	N4	Female, Dutch
I9	P9	Female, Dutch	N9	Female, Dutch
I10	P10	Male, Dutch	N10	Female, Dutch
I11	P11	Female, Surinam (Dutch)	N11	Male, Dutch
I12	P12	Female, Italian (English)	N12	Male, Dutch
I13	P13	Female, Antillean (Dutch)	N13	Female, Dutch
I14	P14	Female, Dutch	N2	Male, Dutch
I15	P15	Male, Dutch	N10	Female, Dutch

a Interview language is mentioned for the non‐Dutch native speakers. With native speakers, interview language was Dutch.

Strikingly, patients remembered the course of events in a detailed manner, despite having severe psychiatric symptoms. They sometimes even remembered events in more detail than the involved nurse. For example, the following patient stayed in the seclusion room from Monday until Thursday:

P1: I come inside, the police arrives, they take me in there. I went crazy, they give me an injection. Another injection, without without any … give me an injection, I stay in there from Monday to Thursday

N1: And sir was taken into the seclusion room under coercion, there he is administered an intramuscular antipsychotic and a new medication policy was dictated. And sir stayed, I think, about a week in the seclusion room

#### 3.2.2 Subjective experience

The subjective experience of patients and nurses regarding the aggressive incident differed in most cases:

P12: So, I spit on the, like I do tuff. But not on him, on the ground. And I also clean this; it is not a problem you know. Like a spit and say: ‘what the …., stuff like that. But I did not touch nobody.

N12: At that moment she started to clear her throat, seriously, I saw the spittle on her tongue, so she could spit at me. And the only thing I could do was: push her away

We interpreted differences in subjective experience as a difference in PS of the aggressive incident. We defined PS as “the subjective severity of aggressive behaviour perceived by the aggressor, victim or witness of an aggressive incident”. PS is a construct described in literature around school bullying (Chen, 2015), but was never included in literature on aggression in mental health care. In general, patients perceived the severity of the aggressive incident as lower than nurses:

P5: Yeah, they tell me that I am sexual aggressive with the people but if I took you by arm and say: ‘Come on let's go’. I guess that, yeah, we are two adults and we can have some fun together without any other problems. But of course, if you say: ‘’No, I don't want it’ and I respect you.

N6: It came out of nowhere actually. I entered the corridor and that gentleman comes out of his room and he rushes at me and grabs me and fondles me like this and then he said ‘you are coming with me now’, in English. So he wants to take me, like, to his room. So I said: ‘no, you have to let go of me now. … He says: ‘yes, I am just going to have sex with you now’.

We found several patients that challenged the appropriateness of the response of nurses to the aggressive incident. This is not surprising, based on the difference in PS. We perceived this for instance in the following two examples:

P5: Of course my point of view is very disappointing because I don't make nothing bad and the separation room, I can tell you it is something that is truly terrible

N6: But yeah, still, if you inject him, you still have that the danger. … So, you have to choose for safety so it was decided to bring him to the seclusion room for a continuous stay

P3: I was already tired. I was in my room, getting ready to sleep. I heard extremely loud TV and washing‐up and this and that. At half past two the TV was on, someone was doing the dishes and whatever. Yes and then I did not snap, but I said: ‘come on, I want to sleep’. And then all day he came, that tall bold guy, he came all night with his flashlight and: is he sleeping, is he sleeping. Yeah and then I woke up again.… Well and then I went crazy and they came with 30. … Yes, no, but yes no but they came to the seclusion room. I sat there for a while, three, four hours and then I could go back. But the way it happened, that is just ridiculous.

N3: I suggested….: go to sleep and, as for us, take medication when necessary. That will help you, it is really hard on you to be here like this right now. He refused that, over time. He was insulting in his reactions, threatening: ‘well, you can go get some of those big Ajax [well‐known Dutch soccer team] guys, they'll just smash the door’, those were the kind of things that were said. Towards [female nurse], he was sexually disinhibited, openly horny, to put it like that. He did go to his room for a while and then he woke up again. … And, over time, when he started to bang on the door more, I pressed the alarm. Of course others had already been notified about the situation. Security again, who also responded to the alarm, at that moment no less than three security guards, so six people on staff. At that moment in time we had already umm decided to go give an injection in the seclusion room.

### 3.3 Recommendations

#### 3.3.1 Patients' recommendations

Patients often mentioned personal de‐escalation techniques that were only suitable for themselves, such as music (P6: “Playing the cello calms me down”) and sports (P6: “I need sports, I need some activities and if I have my activities I am relaxed”). This concept is referred to as “meaningful daily activities”. Some patients mentioned personal de‐escalation interventions that were not realistic on the ward, but also gave usable alternatives (P13: “I would like them to build a pool there. You know why? If you are aggressive, you are warm. You must cold water there. If I am aggressive, I go straight to my room and shower. With that cold water I stand like tsjoeh”). Some patients gave recommendations that seem to be highly affected by psychiatric symptoms, especially when patients suffer from paranoia and anxiety, such as a patient that assumed (wrongly) the staff had “paralyzers” (P4: “You have paralyzers. You could have used them when he had Anthrax. You could have paralyzed him instead of inject him”). Patients frequently expressed their wish for more humanity (P12 “Be humane. Think and think one moment, maybe she is angry for this so let's solve her problem”) and freedom (P1: “I want to have my freedom, even a bit. That is what I want to have”) during involuntary admission. This subtheme emerged especially with patients by who the incident resulted in coercive measures (P5: “You can't give medicine if I don't want it, it is a truly big violence and it's also against my human rights”). Another subtheme was interpersonal contact as a method for de‐escalation. Some patients felt like that nurses used coercion too fast and believed that talking would have helped to de‐escalate the incident (P12: “When I say something, say something back to me. But don't grab me”).

Some aggressive incidents ended with staff using coercive measures. During these measures, the patient's autonomy is diminished. Patients advised to respect their autonomy as much as possible, even in the context of coercive measures. Patients expressed the need to take part in the decision of using coercive measure, for example how it is conducted and how long it must last (P9: “The main thing is that you have to take someone out of seclusion as soon as possible, when that person has calmed down again and has come to his senses”) or the use of own clothes for more privacy during seclusion (P13: “And if maybe I don't want to wear that dress. Everybody is looking because you have that mirror and behind those people are standing there to look at you and there is a camera too”).

Ward routine can be described by the daily practice which patients encounter, which is a result of the organizational structure of the hospital. Examples like ward rules and changes in surroundings were mentioned as influential for aggressive incidents. Patients also gave recommendations on how to change ward routines (P14: “I think I went to several rooms, which made me even more confused”).

During the interviews, it was clear that patients were able to give usable recommendations for the prevention of aggressive incidents. However, it seemed important that the interviewer took time to listen and ask comprehensive questions to patients. Due to (sometimes) highly incoherent language of patients, time was needed to gain valuable recommendations. Two patients could not provide coherent or feasible recommendations, from the perspective of the authors.

#### 3.3.2 Nurses' recommendations

Nurses frequently advised the use of medication to prevent aggressive incidents. The rationale is that pharmacological intervention, even pro re nata (PRN) or forced medication, is less coercive than seclusion or restraint. Adequate timing of interventions is critical for de‐escalating aggressive incidents (N10: “So, I don't know if the shift before me, the evening shift, might have noticed and could have given medication earlier or something”).

The recommendations of nurses around the timing of interventions is mostly to start earlier with PRN‐medication or to make contact before the situations escalates. Most nurses who give recommendations on timing are not sure whether this could have de‐escalated the aggressive incident (N9: “To get her out of the garden earlier. Yes. But I don't think it would have caused less aggression”).

Some nurses gave the recommendation related to the facility, such as availability of secured rooms on the ward (other than the seclusion room) and new development of a high intensive care unit (HIC) (Bierbooms, Lorenz‐Artz, Pols, & Bongers, 2017), where the ward will contain separate rooms for one‐on‐one patient care (N5: “….in a future HIC we can go into a separate room, then your social contribution will increase. You can offer more”).

## 4 DISCUSSION

Our main finding is that most patients and nurses described similar facts of aggressive incidents, but differences in the PS. An intervention to respond to aggressive incidents is chosen based on several aspects, among which the severity of the incident. Differences in the interpretation of the severity are likely to result into differences in the interpretation of the appropriateness of the response of nursing staff to the incident. This finding answers our first research question and adds to the literature that patients were more likely to find interventions used after aggressive incidents excessive (Frueh et al., 2005; Whittington & Wykes, 1996).

Severity is found to be a subjective construct which can highly differ between patients and staff. Most patients perceived a lower severity of aggression than nurses. This finding is new in comparison to previous studies that triangulated around the same incident (Ilkiw‐Lavalle & Grenyer, 2003; Omerov et al., 2004). A study into the perception of the social environment at acute psychiatric inpatients wards explained observed differences by contradictory opinions of patients and nurses concerning high staff control and high autonomy (Schjodt, Middelboe, Mortensen, & Gjerris, 2003). The authors explained the difference in staff control by suggesting that staff tend to underestimate the extent to which they use limiting and controlling measures towards patients. Differences in perception of the severity of an incident might have implications for future therapeutic alliance between patient and nurse (Höfer et al., 2015). For example, patients could experience the nurses' intervention as disproportionate as they wish to maintain their autonomy.

Although many studies have been performed to reduce the use of coercive measures in psychiatric wards (Bowers et al., 2015), coercive measures still occur. Our study shows that exploring the PS of coercive measures might be a starting point to restore the contact with the patient. Subsequently, discussing differences in perspective may improve the therapeutic alliance after an aggressive incident.

Regarding recommendations, one new concept emerged namely personalized de‐escalation interventions. This study adds an in‐depth evaluation of the exact difference in perspectives of patients and nurses after aggressive incidents. Furthermore, it suggests that it is valuable to explore differences in perspectives, in particular PS, after aggressive incidents and ask both parties for recommendations on improvement of care. We found that most inpatients on a closed psychiatric ward were capable and willing to give recommendations regarding safety and de‐escalation. Examples of types of recommendations are *meaningful daily activities, humane treatment and involvement of patients in decision‐making around coercive measures*. These findings are in line with previous studies (Gillig et al., 1998; Gudde et al., 2015; Ilkiw‐Lavalle & Grenyer, 2003; Kontio et al., 2014, 2012; Meehan et al., 2006). Similar to previous research, substantial difference between patients and nurses in concepts of recommendations emerged (Hallett et al., 2014). Earlier research showed that evaluation of an aggressive incident is possible within 2–7 days after the incident (Kontio et al., 2012). Our study is in line with the finding that it is useful to interview both patients and nurses shortly after an incident (Ilkiw‐Lavalle & Grenyer, 2003). The benefits of interviewing shortly after an incident are that complementary recommendations are yielded and can be applied directly. Most of the recommendations of patients were highly personal and not covered by nurses. It is plausible that the validity of patients' recommendations obtained in our study is substantial, because patients were asked while they were in a comparable psychopathological state as during the incident.

### 4.1 Limitations

This is, to our knowledge, the first qualitative study that evaluated perspectives about a specific aggressive incident shortly after the incident by comparing perspectives of patients and nurses. Behaviourally disturbed patients who are involuntarily admitted are generally difficult to include in research. They often refuse consent or a lack of decisional capacity to give informed consent is assumed (Lopez‐Jaramillo, Tobler, Gomez, & Triana, 2016; Parmigiani et al., 2016). Our results show that, although suffering from a severe mental condition, most such patients are willing and able to participate in a qualitative study.

Some limitations need to be considered while interpreting the results. Since it was a mono‐centre study, unique characteristics may have influenced perspectives of participants. The interviews were performed a median of 3 days after the incident, but two of the interviews were performed substantially later (9 and 13 days after the incident). All patients were admitted when the interviews were conducted. Therefore, social desirability influencing their answers or recommendations should be considered as a potential limitation.

During the study preparations were performed to adapt to a HIC model (Bierbooms et al., 2017) and this may have influenced perception of nurses. Further, because this study evaluated mostly severe incidents, it is possible that this influenced the participants in their perspective and recommendations. Evaluation of minor incidents needs further research.

## 5 CONCLUSION

The perspective of patients is essential for improving quality and safety of care (Pincus et al., 2007). However, providing care that is respectful and responsive to individual patient preferences can be challenging in case of involuntary admission (Pincus et al., 2007). This study shows that incorporating perspectives of psychiatric inpatients who act aggressively, seems feasible and may be useful to improve quality and safety. A previous study reported that staff had more opportunities to debrief than patients (Ilkiw‐Lavalle & Grenyer, 2003). We recommend, in line with previous research, to evaluate aggressive incidents at closed psychiatric wards with patients and staff (Bensley, Nelson, Kaufman, Silverstein, & Shields, 1995; Bonner, Lowe, Rawcliffe, & Wellman, 2002; Gillig et al., 1998; Ilkiw‐Lavalle & Grenyer, 2003). Our findings of a common ground in all incidents (factual course of events), could serve as a starting point for debriefing. We argue that PS of aggression and the decision‐making process leading to interventions are important concepts to discuss with patients and staff.

To compare different views, we recommend that debriefing should be held by independent staff members. Since the recommendations of patients and nurses are repeatedly found to be complementary, it is advisable to debrief both (Hallett et al., 2014). Regarding the theoretical method of debriefing, previous studies in other settings showed that technical debriefing (i.e., not focussing on feelings but on facts) improve the outcome of patients after psychological trauma (Sijbrandij, Olff, Reitsma, Carlier, & Gersons, 2006). Rapid quality cycles can be used to enhance and evaluate implementation of debriefing into practice (Etchells, Ho, & Shojania, 2016). Patients and staff members should collaborate in identifying strategies to prevent dangerous situations in the future, to reduce the chance of using coercive measures (Stewart, Van der Merwe, Bowers, Simpson, & Jones, 2010). An example is to capture patients' personal crisis management strategies in a shared crisis management plan. Patient safety plans might provide a framework to put this into practice (Jonikas, Cook, Rosen, Laris, & Kim, 2004). When debriefing takes place shortly after an incident, a sense of control and autonomy could be restored. Ultimately, the evaluation of past aggression might prevent new aggressive incidents, thereby prevent the use of coercive measures and contribute to making the psychiatric inpatient unit a safe place for everyone.

## CONFLICT OF INTEREST

No conflict of interest has been declared by the authors.

## AUTHOR CONTRIBUTIONS

JMV, PD, LdH made substantial contributions to conception and design, or acquisition of data, or analysis and interpretation of data; JMV, PD, LLB, BS, CHML, LdH involved in drafting the manuscript or revising it critically for important intellectual content; JMV, PD, LLB, BS, CHML, LdH given final approval of the version to be published. Each author should have participated sufficiently in the work to take public responsibility for appropriate portions of the content; agreed to be accountable for all aspects of the work in ensuring that questions related to the accuracy or integrity of any part of the work are appropriately investigated and resolved.
