# Supplementary material for: “But I did not touch nobody!”—Patients' and nurses' perspectives and recommendations after aggression on psychiatric wards—A qualitative study
Source: J Adv Nurs. 2019 Jul 10;75(11):2845–54. doi: 10.1111/jan.14107 (PMC6899923; doi:10.1111/jan.14107)
Supplement: Supplementary file 1 [file JAN-75-2845-s001.pdf]

Academisch Medisch Centrum
Universiteit van Amsterdam

Aan de heer prof. dr. L. de Haan
Psychiatrie
PA0-150

Amsterdam, 9 december 2015
uw kenmerk:
ons kenmerk: W15_336 # 15.0391
betreft:

Medisch Ethische Toetsingscommissie
E2-172
doorkiesnummer: 566 7389

Uw brief: **Perspectieven en suggesties van psychiatrische patiënten na agressieve incidenten op een psychiatrische IC**

Geachte heer De Haan,

Uw brief d.d. 27 november 2015 betreffende bovengenoemde studie is op 8 december jl. besproken in de vergadering van het dagelijks bestuur.

Het dagelijks bestuur is van oordeel dat bovengenoemde studie niet valt binnen de reikwijdte van de Wet medisch-wetenschappelijk onderzoek met mensen, aangezien er geen sprake is van wetenschappelijk onderzoek zoals bedoeld in artikel 1, eerste lid onder b van de WMO, daar de proefpersonen niet aan handelingen worden onderworpen, en/of aan de proefpersoon wordt geen bepaalde gedragswijze opgelegd.

Een formele beoordeling door onze commissie is derhalve niet noodzakelijk.

Met vriendelijke groet,
namens de Medisch Ethische Toetsingscommissie

Mw. T. Groenveld
ambtelijk secretaris

Bijlage: verklaring in het Engels (z.o.z.)

c.c. per email: j.m.vermeulen@amc.uva.nl; p.doedens@amc.uva.nl

Meibergdreef 9 Postbus 22660 1100 DD Amsterdam T 020 5669111 F 020 5664440 www.amc.nl

To whom it may concern,

Referring to our letter of December 9, 2015 (reference number W15_336 # 15.0391) we are pleased to confirm that the Medical Research Involving Human Subjects Act (WMO) does not apply to the above mentioned study and that an official approval of this study by our committee is not required.

Yours sincerely,
on behalf of the Medical Ethics Review Committee of the Academic Medical Center,

Mrs. T. Groenveld
secretary
