# Supplementary material for: “But I did not touch nobody!”—Patients' and nurses' perspectives and recommendations after aggression on psychiatric wards—A qualitative study
Source: J Adv Nurs. 2019 Jul 10;75(11):2845–54. doi: 10.1111/jan.14107 (PMC6899923; doi:10.1111/jan.14107)
Supplement: Supplementary file 2 [file JAN-75-2845-s002.docx]

**Supplement 1 Coreq Checklist**

**Domain 1: Research team and reflexivity**

*Personal Characteristics*

1) Interviewer/facilitator: JV (Sample/Participants)

2) Credentials: MD (Authors)

3) Occupation: PhD-student (Data analysis)

4) Gender: Female (Data collection)

5) Experience and training: Medical training and additional training in qualitative research (Data analysis)

*Relationship with participants*

6) Relationship established: No

7) Participant knowledge interviewer: No

8) Interviewer characteristics: Explained in section data analysis

**Domain 2: study design**

*Theoretical framework*

9) Methodological orientation &theory: Grounded theory, as stated by Corbin & Strauss (Design)

*Participant selection*

10) Sampling: Convenience sample (Sample/Participants)

11) Method of approach: Face to face, the researcher came to the ward (Sample/Participants)

12) Sample size: Fifteen patients and thirteen nurses fifteen unique aggressive incidents, total of thirty-one interviews (Sample/Participants)

13) Non-participation: Seven patients refused to participate mostly because of lack of interest or less frequently because of a lack of trust in audiotaping their comments (Sample/Participants)

*Setting*

14) Setting of data collection: On the ward, nurses were occasionally interviewed by telephone (Data collection)

15) Presence of non-participants: No

16) Description of sample: Table 1 (patients), table 2 (patient-nurse dyads, incidents), section Sample/Participants

*Data collection*

17) Interview guide: There were questions (3) which were pilot tested (Data collection)

18) Repeat interviews: No

19) Audio/visual recording: Audiotaped (Data collection)

20) Field notes: No, because of contra-indication, as explained in the section Data collection

21) Duration: The interviews lasted from eight to twenty-five minutes with an average duration of fourteen minutes (Findings)

22) Data saturation: Yes, as explained in the sections Data analysis and Findings

23) Transcripts returned: No, deemed infeasible. From experience we now large loss to

follow-up in this vulnerable group (Data analysis)

**Domain 3: analysis and findings**

*Data analysis*

24) Number of data coders: 2 (Data analysis)

25) Description of the coding tree: Upon request (Data analysis)

26) Derivation of themes: In coding tree upon request (Data analysis)

27) Software: MaxQDA (Data analysis)

28) Participant checking: No, because from experience we now large loss to follow-up in this

vulnerable group (Data analysis)

*Reporting*

29) Quotations presented: Yes (Findings)

30) Data and findings consistent: Yes (Findings)

31) Clarity of major themes: Yes, as explained in the section Findings, subsection Concepts

32) Clarity of minor themes: Yes, as explained in the section Findings, subsection Perspectives and Recommendations
